# Assessing the Quality of Burkina Faso Soybeans Based on Fatty Acid Composition and Pesticide Residue Contamination

**DOI:** 10.3390/molecules27196260

**Published:** 2022-09-23

**Authors:** Elisabeth Rakiswendé Ouédraogo, Kiessoun Konaté, Abdoudramane Sanou, Hemayoro Sama, Ella Wendinpuikondo Rakèta Compaoré, Oksana Sytar, Adama Hilou, Marian Brestic, Mamoudou Hama Dicko

**Affiliations:** 1 Laboratory of Biochemistry, Biotechnology, Food Technology and Nutrition (LABIOTAN), Department of Biochemistry and Microbiology, University Joseph KI-ZERBO, Ouagadougou 09 B.P. 848, Burkina Faso; 2 Applied Sciences and Technologies Training and Research Unit, Department of Biochemistry and Microbiology, University of Dedougou, Dedougou 09 B.P. 176, Burkina Faso; 3 Laboratory of Biochemistry and Chemistry Applied (LABIOCA), University Joseph KI-ZERBO, Ouagadougou 09 B.P. 848, Burkina Faso; 4 Institute of Plant and Environmental Sciences, Slovak Agricultural University in Nitra, 94976 Nitra, Slovakia

**Keywords:** soybean, fatty acid, pesticides residues, LMRs

## Abstract

Soybean is widely used in the food industry because of its high fatty acid and protein content. However, the increased use of pesticides to control pests during cultivation, in addition to being a public health concern, may influence the nutritional quality of soybeans. This study aimed to assess the nutritional quality of soybeans with respect to fatty acid profile and pesticide residue contamination. The levels of fatty acids and pesticides in soybean varieties G196 and G197 were determined by gas chromatography and by the QuEChERS method, respectively. The results showed a significant variation in the quantitative and qualitative fatty acid composition of the two varieties, with 18.03 g/100 g and 4 fatty acids detected for the G196 variety and 21.35 g/100 g and 7 fatty acids for the G197 variety, respectively. In addition, 12 active pesticide compounds were found, and among them, imazalil, quintozene, cyfluthrin and lindane exceeded their maximum limits. The G197 variety had a better nutritional profile compared to G196. The profile of fatty acids and the content of pesticide residues were used as important determinants for soybean utilization in human nutrition.

## 1. Introduction

Soybean (*Glycine max*) is a highly consumed food in the world with an annual production of 348.7 million tons [1]. In 2019, Brazil and the United States of America were the largest producers, with productions of 114.27 and 96.8 million tons, respectively [2]. In 2021, the European Union cultivated about 2.7 million tons of soybean [3]. Soybean is still a minor crop in the central and northern growing regions of Europe and many countries in the world, even if there is a high demand for it. In Burkina Faso, the cultivation of soybeans, in particular the varieties G196 and G197, is booming, with production exceeding 51,708 tons in 2020 [4]. Indeed, soybean is a highly nutritional plant providing a significant supply of vegetable proteins, carbohydrates, dietary fibers, vitamins, minerals, bioactive compounds [5] and fatty acids (FAs), including two saturated FAs (palmitic acid (16:0) and stearic acid (18:0)), and three unsaturated FAs (oleic acid (18:1), linoleic acid (18:2) and α-linolenic acid (18:3) [6]. Soybeans contain 20–30% fat and account for 29% of the world’s vegetable oil supply. In addition to a food role, the FAs of soybean (C16:0; C18:0; C18:1; 18:2 and 18:3), in particular *w3* and *w6*, are of medicinal interest, because they play a role in reducing the levels of VLDL cholesterol in the blood [7]. Linolenic acid, in particular, also helps to reduce the incidence of diabetes mellitus and hypertension [7]. Palmitic acid, associated with stearic acid, has antimicrobial properties against *Staphylococcus aureus* and *Helicobacter pylori*, the latter against *Streptococcus pyogenes* [8]. In addition to its food and pharmaceutical properties, the soybean content in oils and the FA composition of this oil, are good candidates for the production of biodiesel [9]. Several parameters, such as genetic variability and cultivation conditions [10], including the use of pesticides, can influence the nutritional quality of soybeans. For example, the incorrect disposal of seeds treated with pesticides can be a risk to the permanence of life. The chromatographic analysis estimated the presence of carbendazim above the maximum residue limits established by legislations [11]. Therefore, a validation of the nutritional quality of soybeans should be correlated with their low composition of toxic contaminants, such as pesticides. Pesticides in food cannot be ignored because of their acute adverse effects, such as nausea, headaches, cancers, reproductive damage and endocrine disruption [12]. To this end, an assessment of the state of pesticide contamination of food products requires knowledge of maximum residue limits (MRLs), which are the maximum level of residues expected in a food product when a pesticide is used as intended [13]. Specific to each pesticide active molecule, these food residues are set below any levels of toxicological risk to humans, as recommended by the World Health Organization [14]. In addition to these toxicological risks, the use of plant protection products can significantly affect the chemical composition of soybeans [12]. Recently, it has been shown that fatty acid composition is a candidate indicator to determine the response to pesticides [15]. Nowadays, the “quick, easy, cheap, effective, rugged, and safe” (QuEChERS) method is an accepted analysis and extraction of pesticides in plant materials [16], especially in different plant extracts, such as vegetables containing high amounts of oils [15]. In 2022, Tong et al. combined the advantages of QuEChERS and gas and liquid chromatography high-resolution mass spectrometry to analyze fatty acid compositions and levels of pesticide residues [17]. It was confirmed that such a combination of analysis methods is suitable for the pesticide multiresidue determination of soybean-based beverages in routine laboratory analyses. The presented study aimed to assess the nutritional quality of two soybean varieties widely grown and consumed in Burkina Faso, through the fatty acid profiling and pesticide analysis, using gas chromatography and the QuEChERS method.

## 2. Results

### 2.1. Fatty Acids Analysis

The FAs profile is an important factor in the nutritional quality of edible oils. The extraction of oil revealed that G197 has a higher total lipid content (21.35% ± 0.18) than G196 (18.03% ± 0.05). In both oils, among the detected FAs (i.e., pentadecanoic, palmitic, stearic, palmitoleic, linoleic, γ-linolenic, eicosatrienoic, erucic and docosadienoic acids), the G196 variety displayed more saturated FAs than the G197 variety. In contrast, the G197 variety contained more unsaturated FAs (linoleic acid, γ-linolenic acid, eicosatetraenoic acid, erucic acid) than G196 (γ-linolenic acid). For the quantified FAs (pentadecanoic acid, palmitic acid, γ-linolenic acid) in both varieties, G196 showed a higher level. Two of the main soybean FAs, stearic acid (C18:0) and oleic acid (C18:1) were not quantified in any of the varieties. In this study, soybean genotype had a strong influence on the quality and content of FAs (Table 1).

### 2.2. Pesticides Residues

**Figure 1:**
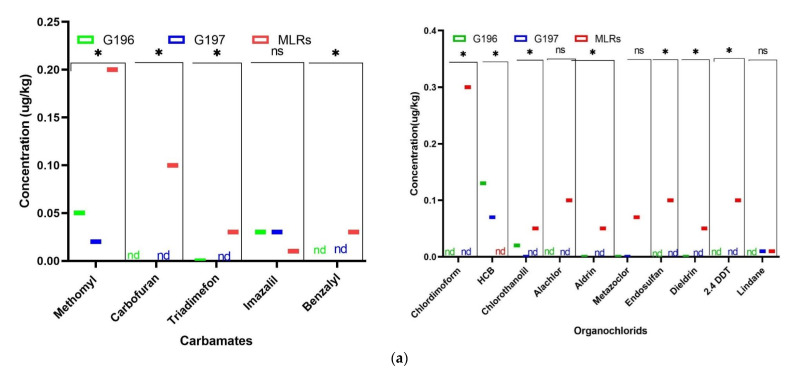

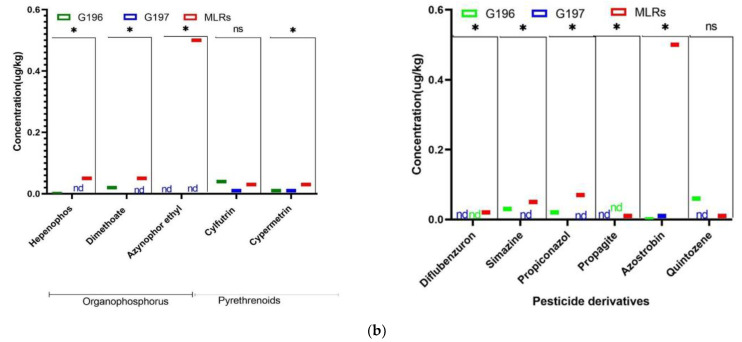
Comparison of pesticide levels in soybean against MRLs (**a**,**b**). Values are represented as mean ± SEM; “*” indicates a significant difference for each line (*p* < 0.05); “ns” indicates a significant difference for each line (*p* < 0.05).

The QuEChERS method is a powerful tool for the analysis of pesticides in complex fat matrices. Based on the demonstrated acceptable accuracy and precision, LOQ levels ranged from 0.002 mg/kg to 0.005 mg/kg (Table 2). The analysis of pesticide residue contamination is an important factor in soybean quality. Chromatographic analysis allowed for the detection of four main families of pesticides i.e., organophosphates (OP), organochlorines (OC), carbamates (C) and *pyrethroid* (P), as well as triazin, triazol, benzoylureas, and strobin (D) derivatives in the soybean samples studied (Table 2). Among the 26 active molecules screened, 12 molecules were detected and quantified in one or both soybean varieties, namely methomyl, HCB, dimethoate, simazine, quintozene, lindane, chlorothalonil, propiconazole, imazalil, cyfluthrin, cypermethrin and azoxystrobin. Among these molecules detected, the organochlorine active ingredients are in the majority in both varieties, and at various concentrations. In this respect, the different contents of the pesticide families allowed for the increasing classification of the detected families: OC >D> OP> C for both varieties. Unfortunately, it appears from the study that only the pesticides methomyl, dimethoate, imazalil and *azoxystrobin* are authorized by the Sahelian Pesticide Committee (SPC). Nevertheless, according to the process of absorption and accumulation of pesticide residues, the detected and quantified molecules all showed levels well below the maximum residue limits, with the exception of imazalil, quintozene and cyfluthrin for the G196 variety and imazalil and lindane for the G197 variety (Figure 1a,b).

## 3. Discussion

Fatty acids are made up of saturated and unsaturated fatty acids, which are further divided into monounsaturated fatty acids (MUFA) and polyunsaturated fatty acids (PUFA) [18]. The latter are the most important nutritional components of edible oils or other functional foods [19]. Many previous studies have shown that soybeans contain various nutritional components beneficial to human health due to their primary metabolites, such as proteins, lipids, carbohydrates and many bioactive compounds [20]. Indeed, soybeans are a highly lipid-rich nutritional grain. Their fats have an exceptionally high content of PUFA, such as linoleic (C18:2) and γ-linolenic (C18:3) acids, and also significant amounts of MUFA, such as oleic acid (C18:1), and moderate amounts of saturated FAs, such as palmitic (C16:0) and stearic (C18:0) acids [21]. The geographical origin of the genotype affected seed fatty acid composition, indicating that in each ecological region, genotypes tend to have a different composition [18]. The biosynthesis of nutrients in soybean, especially FAs, is influenced by various factors, including variety (genotype), maturity, soil, climatic, growing conditions and biotic and abiotic stresses etc. [22]. This study revealed that genetic variability had a strong influence on qualitative FAs content. A significant difference in lipid level was found between the two soybean varieties, with G197 displaying the highest content. Due to the diversity of crops, as a consequence of the adaptation to complex climatic and soil environments, the germplasm influences the nutritional quality of the soybean, including the lipid profile [10]. The current study also revealed a high diversity of PUFA: low concentrations in G197, and higher concentrations in G196, notably higher in ɣ-linolenic acid. Indeed, biochemical composition would indicate that in soybeans and other plant species, the synthesis of PUFA is an inherited trait [23]. Contrary to previous data, our study did not unambiguously detect some FAs (oleic acid, stearic acid and α-linolenic acid) usually found in soybeans. These results confirm the influence of the germplasm on the fatty acid content of the seeds, which has been explained by the duration of seed filling, including the time to maturity [10,11,12,13,14,15,16,17,18,19,20,21,22,23,24]. In 2019, Seym et al. found the highest concentrations of linoleic acid (C18:2) in industrially processed soybean oils, compared to myristic acid (C14:0), palmitic acid (C16:0), palmitoleic acid (C16:1), stearic acid (C18:0), oleic acid (C18:1), and linolenic acid (C18:3) [25]. In our studies on varieties G196 and G197, there were different results regarding some PUFA, such as linoleic acid (C18:2), which was absent in G196, and also γ-linolenic acid. Both varieties G196 and G197 showed the presence of pentadecanoid acid, and its concentration depended on variety. Pentadecanoid acid was present in the soybean of the imported soybean variety G196, but absent in the soybean of variety G197 grown in Burkina Faso. In 2021, Szpunar-Krok and Wondołowska-Grabowska showed that soybean fat quality indices were significantly influenced by cultivar type [26]. For the evaluation of the effect of cultural practices on the nutritional quality of soybeans, the molecular characterization of pesticides allowed for the detection of the four main families of pesticides: carbamates, orgnanochlorines, organophosphates, pyrethroids, as well as triazines, strobin and benzoylurea derivatives. These results showed a diversified use of pesticides in the soybean crop. The non-specific use of pesticides in soybean cultivation corroborates those findings of Lehman et al. (2017), in vegetable cultivation in Burkina Faso [27]. The inappropriate use of these pesticides, including organochlorines, pyrethroids and organophosphates, may create long-term public health risks [28]. The research analysis of both experimental soybeans of the original soybean varieties G196 and G197 assessed the presence of organochlorine pesticide residues at various concentrations. Organochlorine pesticide residues were detected in soybean vegetables collected in Chinese markets as well [29]. In particular, the mechanistic analysis of organochlorine-induced dementia has suggested that the high bioaccumulation of pesticides in brain fat can lead to oxidative stress and neuroinflammatory responses, altered Ca^2+^ homeostasis, neurotransmitter dysfunction and the progressive loss of neurons and synapses [30]. Intensive exposure to these active ingredients can have profound effects on human health, including the increased risk of cancer, diabetes, genetic disorders and neurotoxicity [31]. It interferes with normal hormonal function and has negative effects on the reproductive system [32]. The chromatographic analysis of the pesticides showed that the 26 targeted active pesticide molecules were detected and quantified in one or both varieties. Of these detected pesticides, only methomyl, chlorothalonil, cypermethrin and azoxystrobin were authorized by the Sahelian Pesticide Committee. These results confirm a cross-border traffic of unauthorized pesticides which could have serious consequences on human health. The presence of carbendazim above the maximum residue limits was observed in the soybean seeds of Brazilian varieties [11]. Nevertheless, these active molecules were found to be significantly below the maximum residue limits, with the exception of imazalil, cyfluthrin, lindane and quintozene. In addition, variety G197 showed a lower pesticide uptake compared to the G196 variety. This variety, G197, thus presents the best nutritional profile because it is rich in PUFA and has a low pesticide residue content. In this respect, several hypotheses suggest a correlation between the fatty acid profile and pesticide residues. Among hypotheses that could explain the metabolic disorder of fatty acids by pesticides, it can be noted that: (i) the exposure of leaves to pesticides would inhibit the biosynthesis of very long chain fatty acids (VLCFA) in plant cells; and (ii), the inhibition of the multi-enzyme system acyl-CoA elongase [33]. For the first hypothesis, de novo FAs biosynthesis in higher plants is catalyzed by a battery of enzymatic steps (fatty acid synthase) in the plastids. Thus, long-chain fatty acids (LCFAs) are synthesized by the sequential addition of C2 moieties to C18 FAs and then incorporated into the main lipid pools (triacylglycerols, waxes, phospholipids, sphingolipid complexes) [34]. During this synthesis, exposure to pesticides, particularly organochlorines, induces the inhibition of cell division and hence VLCFA synthesis [34]. Some authors assessed that herbicides were potent inhibitors of acetyl-CoA carboxylase in the barley chloroplast [35]. A physiological approach would show symptoms of the pigmentation of the fronds followed by tissue necrosis; these symptoms are typical of inhibitors of VLCFA synthesis and cell division [36]. The results of this study indicated the need to include official controls on pesticide residues in soybean varieties used for safe food production.

## 4. Materials and Methods

### 4.1. Experimental Material

The biological material consisted of soybeans (*Glycine max*) harvested in 2019 from farmers in Manga (11°39′48″ N, 1°04′12″ W), in the North Central Region. The two major varieties grown in the country, G196 and G197, were selected for the experiment [37]. The influence of individual farmer cultivation practices was minimized by forming a composite sample using the cluster sampling method [38].

### 4.2. Chemicals and Reagents

Acetonitrile (ACN), acetone, and methanol (MeOH) analytical grade solvents (purity ≥99.5%; Sigma Aldrich, Taufkirchen, Germany) were used for the determination of pesticides and FAs. Reagents, sodium chloride (NaCl), sodium citrate (Na Citrate), 0.5 g of anhydrous sodium citrate (Na_3_C_6_H_5_O_7_), graphite (C18) (purity ≥ 95%; Sigma Aldrich, Taufkirchen, Germany), and MilliQ water from a Millipore purification system were used for pesticide identification, and potassium hydroxide (KOH) for lipid profiling. The standard pesticides, methomyl, propoxur, carbofuran, difluorobenzamide, heptenophos, chlorodimeform, HCB, dimethoate, simazine, quintozene, lindane, chlorothalonil, alachlor, aldrin, triadimefor, metazachlor, endosulfan, imazalil, dieldrin, benalaxyl, propiconazol, 2,4-DDT, propargite, azynophor ethyl, cyfluthrin, cypermethrin and, azoxystrobin were obtained from an analytical laboratory (Ouagadougou, Burkina Faso). Standard stock solutions of pesticides were prepared at 1000 mg L^−1^ in ACN, and daily dilutions were performed for the different calibrations. For the FAs profile, the following FAs were tested for: Palmitic acid (C16:0), stearic acid (C18:0), oleic acid (C18:1), linoleic acid (C18:2), γ-linolenic acid (C18:3), lauric acid (C12:0), pentadecanoic acid (C15:0), eicosatrienoic acid (C20:3), and erucic acid (C22:1).

### 4.3. Total Fat Content

Hexane extraction was performed with the Soxhlet apparatus. Total fat was collected after evaporation (Rotavapor) of the hexane, and was used for characterization.

### 4.4. Analysis of FAs by Gas Chromatography

**Figure 2:**
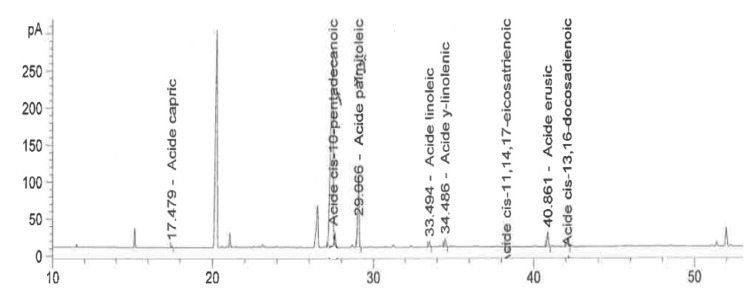
Chromatogram of fatty acid samples (G197).

For FAs analysis, fatty acid methyl esters (FAMEs) were initially prepared from oil extracts by transmethylation, using a methanolic potassium hydroxide solution (4 M CH_3_OH, KOH). In detail, 1 mL of hexane was used to dissolve 50 mg of oil samples extracted from soybeans using a vortex after 1 min. Then, 2 mL of 4 M methanolic KOH was added and the mixture was heated in a water bath for 30 s. Distilled water (1 mL) was added and the organic phase was collected in a vial for injection. Characterization of the fatty acids was carried out using a gas chromatograph (GC Agilent 6890 N, Santa Clara, CA) equipped with a flame ionization detector (FID). During the sample run, 1 µL of FAME sample was injected into the GC-FID and separated using a DB-23 capillary column (60 m × 0.32 mm × 0.25 µm). The oven temperature was successively programmed to 130 °C for 5 min, then to 170 °C at 5 °C/min, to 215 °C at 1.5 °C/min, to 240 °C at 40 °C/min, and this temperature was then constantly maintained for a further 5 min. Helium was used as carrier gas, injector split ratio 1:50, flow rate 1 mL/min. The FAs were individually identified and quantified using the chromatogram peak areas of previously calibrated external standards [39,40]. A methanolic stock solution (1000 mg L^−1^) of the standards (palmitic acid (16:0), stearic acid (18:0), oleic acid (18:1), linoleic acid (18:2), γ-linolenic acid (18:3), lauric acid (12:0), pentadecanoic acid (C15:0), eicosatrienoic acid (20:3), and erucic acid (22:1)) was available, and fresh dilutions for the working solutions were prepared daily (Figure 2).

### 4.5. Pesticides Residues

**Figure 3:**
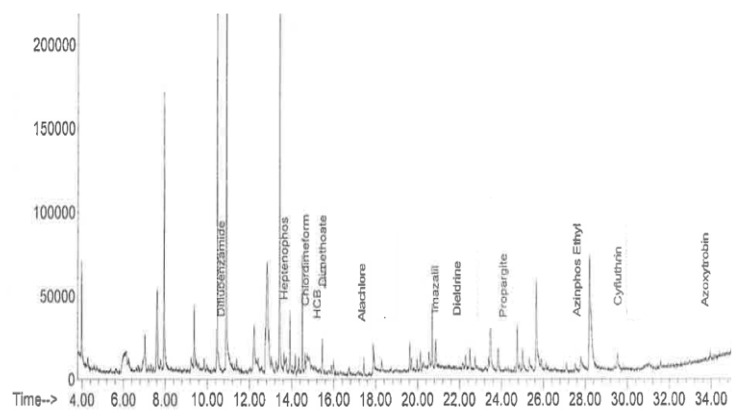
Chromatogram of samples in pesticide residues (G197).

The QuEChERS (Quick, Easy, Cheap, Efficient, Rugged and Safe) method was used, with some modifications, for the determination of pesticide molecules [41,42]. Briefly, the micro-extraction consisted of weighing 5 g of each finely ground sample and solubilizing it in 10 mL of ACN. To this reaction medium was added the extraction reagent consisting of 1 g of NaCl, 1 g of sodium citrate (Na-Citrate), 0.5 g of anhydrous Na-Citrate and 4 g of magnesium sulphate (MgSO4), and the whole mixture was then centrifuged at 3000 rpm for 5 min. The supernatant was purified with salts (anhydrous sodium sulphate) and graphitic carbon (GCB). The purified extract was used for analysis by gas chromatography coupled to mass spectrometer. For this purpose, the chromatograph used was an Agilent 7890A coupled to mass spectrometry (Agilent 5975C inert). The GC system was equipped with an Agilent fused silica capillary column HP-5 ms (30 m × 0.25 mm × 0.25 mm). The chromatographic instrumental settings were as follows: the carrier gas was helium, injector set at 250 °C in split-less mode; the GC oven temperature program was initiated at 50 °C, raised to 100 °C (at a rate of 25 °C/min), and then raised from 100 °C to 300 °C (at a rate of 7.5 °C/min) before being held at this temperature for 3 min; the injection volume was 1 mL, and the flow rates of makeup gas were 20 mL/min. The detector was run in selected ion monitoring mode with the following settings: Squad 180 °C; MS source 230 °C; ion source: EI, 70 eV. Three ions were selected for each pesticide (Figure 3). The highest relative abundant ion was used as the quantifier ion, while the other ions were taken for confirmation as qualifier ions. The system was connected to a computer equipped and controlled by Varianworkstation^®^ software (Agilent Technologies) [43,44]. The limit of detection, limit of quantification and validation data sets were performed in accordance with the European guidelines SANCO/12571/2013 in this study [45].

## 5. Conclusions

This study showed that the biochemical composition of soybeans, with regard to the fatty acid profile, depends on genetic variety. Comparison of the varieties grown in the same environment revealed that the soybean variety G197 contained interesting polyunsaturated fatty acids and had a very low intake of pesticide residues. These findings can be used as important determinants for soybean utilization in human nutrition. Furthermore, it would be interesting to extend the sampling in order to establish a linear regression between the pesticide toxicity threshold and the alteration of the fatty acid profile. Finally, awareness should be maximized among farmers for a responsible use of pesticides and the production of safe food.

## Figures and Tables

**Table 1:** Characterization of fatty acids in two soybean varieties.

Fatty Acid	Chemical Abbreviation	RT (min)	Proportion (%, w/w)	
			G 196	G197
Lauric acid	C12:0	21.46	Nd	nd
Pentadecanoic acid	C15:0	27.63	1.22 ± 0.12 ^a^	0.23 ± 0.14 ^b^
Palmitic acid	C16:0	29.68	1.33 ± 0.33 ^a^	0.23 ± 0.12 ^b^
Stearic acid	C18:0	32.46	Nd	nd
Palmitoleic acid	C16:1 (cis-9)	29.105	2.44 ± 0.12 ^a^	nd
Oleic acid	C18:1 (cis-9)	32.71	Nd	nd
Linoleic acid	C18:2 (cis-9;12)	34.49	Nd	0.28 ± 0.12 ^a^
γ-Linolenic acid	C18:3 (cis-9;12;15)	34.48	1.33 ± 0.22 ^a^	0.28 ± 0.11 ^b^
Eicosatrienoic acid	C20:3 (cis-5;8;11)	38.28	Nd	0.23 ± 0.11 ^a^
Erucic acid	C22:1 (cis-13)	40.86	Nd	0.28 ± 0.13 ^a^
Docosadienoic acid	C22:2 (cis-13;16)	44.26	Nd	0.23 ± 0.13 ^a^
Fatty acid content			18.03 ± 0.05 ^b^	21.35 ± 0.18 ^a^

The values are represented as mean ± SEM; RT: Retention time. Numbers with the same letters in upper score are not significantly different (*p* < 0.05).

**Table 2:** Targeted pesticide residues in the two soybean varieties.

Bioactive Compounds	Family	RT (min)	LOQ	LMR
Methomyl *	carbamate	7.29	0.02	0.2
Carbofuran	carbamate	10.39	0.01	0.1
Diflubenzuron	benzoylureas	10.63	0.02	0.02
Heptenophos	organophosphates	13.64	0.05	0.05
Chlordimeform	organochlorines	14.64	0.05	0.03
Hexacyanobenzen	organochlorines	15.20	0.02	nd
Dimethoate	organophosphates	15.45	0.005	0.05
Simazine	triazine	15.61	0.005	0.05
Quintozene	benzoylureas	15.75	0.02	0.01
Lindane	organochlorines	15.84	0.005	0.01
Chlorothalonil *	organochlorines	16.23	0.5	0.05
Alachlor	organochlorines	17.33	0.005	0.1
Aldrin	organochlorines	18.37	0.005	0.05
Triadimefon	carbamate	18.37	0.005	0.03
Metazachlor	organochlorines	19.27	0.01	0.07
Endosulfan	organochlorines	20.43	0.02	0.1
Imazalil	carbamates	20.82	0.02	0.01
Dieldrin	organochlorines	21.87	0.01	0.05
Benalaxyl	carbamate	23.14	0.005	0.03
Propiconazole	triazol	23.34	0.005	0.07
2,4-DDT	organochlorines	23.59	0.005	0.1
Propargite	sulfites esters	24.08	0.005	0.01
Azinphos-ethyl	organophosphates	27.57	0.005	0.03
Cyfluthrin	pyrethroids	29.56	0.02	0.03
Cypermethrin *	pyrethroids	30.38	0.002	0.03
Azoxystrobin *	strobin	33.7	0.005	0.5

* MLRs: Maximal Limits of Residues, LOQ: Quantitation Limit; authorized by the SPC (Sahelian Pesticides Committee), RT: retention time, 2,4-DDT: Dichlorodiphenyltrichloroethane. Compounds were listed as function of their retention time by gas chromatography analysis.

## Data Availability

The data used to support the findings of this study are available from the corresponding author upon request.

## Sample Availability

Analyzed soybean samples are available from the corresponding author.

## References

[1] Cabezudo I., Meini M.R., Di Ponte C.C., Melnichuk N., Boschetti C.E., Romanini D. (2021). Soybean (*Glycine max*) hull valorization through the extraction of polyphenols by green alternative methods. Food Chem..

[2] FAOSTAT (2021). Seeds Quality and Quantity of Soybean [glycine max (L.) merr.] Cultivars in Response to Cold Stress. http://www.fao.org/faostat/en/-data/QC.

[3] Karges K., Bellingrath-Kimura S.D., Watson C.A., Stoddard F.L., Halwani M., Reckling M. (2021). Agro-economic prospects for expanding soybean production beyond its current northerly limit in Europe. 2022. Eur. J. Agron..

[4] Nignan I. Effets de l’application de la Micro-Dose de NPK et d’Urée sur les Rendements du Maïs (*Zea mays L.*) et du Soja (*Glycine max (L.) Merr.*) et sur le sol au Burkina Faso: Cas des Provinces de la Sissili et du Nahouri. Engineering memorandum, University of Nazi Boni. November, 2017, pp. 1–61. https://bibliovirtuelle.u-naziboni.bf/biblio/opac_css/docnume/idr/environnement2/IDR-2017-NIG-EFF.pdf.

[5] Chen Q., Wang X., Yuan X., Shi J., Zhang C., Yan N., Jing C. (2021). Comparison of phenolic and flavonoid compound profiles and antioxidant and α-glucosidase inhibition properties of cultivated soybean (*Glycine max)* and wild soybean. Plants.

[6] Jiang G.L., Katuuramu D.N. (2021). Comparison of seed fatty and amino acids in edamame dried using two oven-drying methods and mature soybeans. J. Sci. Food Agric..

[7] Harris W.S., Mozaffarian D., Rimm E., Kris-Etherton P., Rudel L.L., Appel L.J., Engler M.M., Engler M.B., Sacks F. (2009). Omega-6 fatty acids and risk for cardiovascular disease: A science advisory from the American Heart Association nutrition subcommittee of the council on nutrition, physical activity, and metabolism; council on cardiovascular nursing; and council on epidem. Circulation.

[8] Zheng C.J., Yoo J.S., Lee T.G., Cho H.Y., Kim Y.H., Kim W.G. (2005). Fatty acid synthesis is a target for antibacterial activity of unsaturated fatty acids. FEBS Lett..

[9] Choi Y.M., Yoon H., Shin M.J., Lee Y., Hur O.S., Lee B.C., Ha B.K., Wang X., Desta K.T. (2021). Metabolite contents and antioxidant activities of soybean (*Glycine max (l.) merrill)* seeds of different seed coat colors. Antioxidants.

[10] Abdelghany A.M., Zhang S., Azam M., Shaibu A.S., Feng Y., Qi J., Li Y., Tian Y., Hong H., Li B. (2020). Natural Variation in Fatty Acid Composition of Diverse World Soybean Germplasms Grown in China. Agronomy.

[11] Oliveira J.C.I., Cardoso A.T., Goulart A.C., Oliveira M.A.C., Santos J.P.V., Goulart S.M. (2022). Determination of Pesticides in Soybean Seeds Incorrectly Discarded Near a Spring of the Paranaíba River, GO-Brazil. Chem. Biodivers..

[12] Claeys W.L., Schmit J., Bragard C., Maghuin-rogister G., Pussemier L., Schiffers B. (2011). Exposure of several Belgian consumer groups to pesticide residues through fresh fruit and vegetable consumption. Food Control.

[13] Wahab S., Muzammil K., Nasir N., Khan M.S., Ahmad F., Khalid M., Ahmad W., Dawria A., Kalyan L., Reddy V. (2022). Advancement and New Trends in Analysis of Pesticide Residues in Food: A Comprehensive Review. Plants.

[14] Nguyen T.T., Rosello C., Bélanger R., Ratti C. (2020). Fate of Residual Pesticides in Fruit and Vegetable Waste (FVW) Processing. Plant.

[15] Gonçalves A.M.M., Rocha C.P., Marques J.C., Gonçalves F.J.M. (2021). Fatty acids as suitable biomarkers to assess pesticide impacts in freshwater biological scales—A review. Ecol. Indic..

[16] Wilkowska A., Biziuk M. (2011). Determination of pesticide residues in food matrices using the QuEChERS methodology. Food Chem..

[17] Tong K., Xie Y., Huang S., Liu Y., Wu X., Fan C., Chen H., Lu M., Wang W. (2022). QuEChERS Method Combined with Gas- and Liquid-Chromatography High Resolution Mass Spectrometry to Screen and Confirm 237 Pesticides and Metabolites in Cottonseed Hull. Separations.

[18] Peng L.P., Men S.Q., Liu Z.A., Tong N.N., Imran M., Shu Q.Y. (2020). Fatty acid composition, phytochemistry, antioxidant activity on seed coat and kernel of paeonia ostii from main geographic production areas. Foods.

[19] Orsavova J., Misurcova L., Ambrozova J.V., Vicha R. (2015). Fatty acids composition of vegetable oils and its contribution to dietary energy intake and dependence of cardiovascular mortality on dietary intake of fatty Acids. Int. J. Mol. Sci. Cvd..

[20] Seo W.D., Kang J.E., Choi S., Lee K., Lee M., Park K., Lee J.H. (2017). Comparison of nutritional components (isoflavone, protein, oil, and fatty acid) and antioxidant properties at the growth stage of different parts of soybean *[Glycine max (L.) Merrill]*. Food Sci. Biotechnol..

[21] Azam M., Zhang S., Qi J., Abdelghany A.M., Shaibu A.S., Ghosh S., Feng Y., Huai Y., Gebregziabher B.S., Li J. (2021). Profiling and associations of seed nutritional characteristics in Chinese and USA soybean cultivars. J. Food Compos. Anal..

[22] Bellaloui N., Mengistu A., Smith J.R., Abbas H.K., Accinelli C., Shier W.T. (2021). Effects of charcoal rot on soybean seed composition in soybean genotypes that differ in charcoal rot resistance under irrigated and non-irrigated conditions. Plants.

[23] Wilson R.F., Burton J.W., Brim C.A. (1981). Progress in the Selection for Altered Fatty Acid Composition in Soybeans. https://acsess.onlinelibrary.wiley.com/doi/abs/10.2135/cropsci1981.0011183X002100050039x.

[24] Bellaloui N., Bruns H.A., Abbas H.K., Fisher D.K., Mengistu A. (2020). Effects of harvest-aids on seed nutrition in soybean under midsouth USA conditions. Plants.

[25] Syed N., Mahesar S.A., Tufail S., Sherazi H., Soylak M. (2019). Quality assessment and safety measurement of different industrial processing stages of soybean oil. Turk J. Food Agric. Sci..

[26] Szpunar-Krok E., Wondołowska-Grabowska A., Bobrecka-Jamro D., Jańczak-Pieniązėk M., Kotecki A., Kozak M. (2021). Effect of nitrogen fertilisation and inoculation with bradyrhizobium japonicum on the fatty acid profile of soybean (*glycine max* (L.) merrill) seeds. Agronomy.

[27] Lehmann E., Turrero N., Kolia M., Konaté Y., Felippe L., Alencastro D. (2017). Science of the total environment dietary risk assessment of pesticides from vegetables and drinking water in gardening areas in burkina faso. Sci. Total Environ..

[28] World Health Organization and United Nations Environment Programme, Endocrine Disrupting Chemicals. Report, ISBN: 978-92-807-3274-0 (UNEP) and 978 92 4 150503 1 (WHO) NLM Classification: WK 102.2012. https://apps.who.int/iris/bitstream/handle/10665/78102/WHO_HSE_PHE_IHE_2013.1_eng.pdf.

[29] Cui Y., Ke R., Gao W., Tian F., Wang Y., Jiang G. (2020). Analysis of Organochlorine Pesticide Residues in Various Vegetable Oils Collected in Chinese Markets. J. Agric. Food Chem..

[30] Soheil S., Saravi S., Reza A. (2015). Potential role of organochlorine pesticides in the pathogenesis of neurodevelopmental, neurodegenerative, and neurobehavioral disorders: A review. Life Sci..

[31] Alengebawy A., Abdelkhalek S.T., Qureshi S.R., Wang M.-Q. (2021). Heavy metals and pesticides toxicity in agricultural soil and plants: Ecological risks and human health implications. Toxics.

[32] Cruz S.S., Magnarelli G., Rovedatti G. (2019). Evaluation of endocrine disruption and gestational disorders in women residing in areas with intensive pesticide application: An exploratory study. Environ. Toxicol. Pharmacol..

[33] Tamagno S., Aznar-moreno J.A., Durrett T.P., Prasad P.V.V., Rotundo J.L., Ciampitti I.A. (2020). Field Crops Research Dynamics of oil and fatty acid accumulation during seed development in historical soybean varieties. Field Crops Res..

[34] Busi R. (2014). Resistance to herbicides inhibiting the biosynthesis of very-long-chain fatty acids. Pest Manag. Sci..

[35] Rendina A.R., Felts J.M. (1988). Cyclohexanedione Herbicides Are Selective and Potent Inhibitors of Acetyl-CoA Carboxylase from Grasses. Plant Physiol..

[36] Grossmann K. (2005). What it takes to get a herbicide ’ s mode of action. Physionomics, a classical approach in a new complexion. Pest Manag. Sci..

[37] Sawadogo Y. Etude de l ’ efficacité biologique de l’herbicide SELEKAM–soya (Fluazyfop-p-butyl 12 g/l EC) sur les adventices du soja (*Glycine max* (L.) Merr.) et ses effets sur les propriétés agrochimiques du sol. Engineering memorandum, University of Nazi Boni. July, 2017, pp. 1–85. https://beep.ird.fr/collect/upb/index/assoc/IDR-2017-SAW-ETU/IDR-2017-SAW-ETU.pdf.

[38] Taherdoost H. (2020). Sampling Methods in Research Methodology; How to Choose a Sampling Technique for Research. Int. J. Acad. Res. Manag..

[39] Bougma A., Sere A., Bazie B.S.R., Sangare H., Ouilly J.T., Bassole I.H.N. (2020). Composition and physicochemical properties of Combretum collinum, Combretum micranthum, Combretum nigricans, and Combretum niorense seeds and seed oils from Burkina Faso. JAOCS J. Am. Oil Chem. Soc..

[40] Séré A., Bougma A., Bazié B.S.R., Traoré E., Parkouda C., Gnankiné O., Bassolé I.H.N. (2021). Chemical composition, energy and nutritional values, digestibility and functional properties of defatted flour, protein concentrates and isolates from *Carbula marginella (Hemiptera: Pentatomidae) and Cirina butyrospermi (Lepidoptera: Saturniidae)*. BMC Chem..

[41] Sanou A., Konaté K., Sama H., Dakuyo R., Diao M., Dibala C.I., Dicko M.H. (2020). Cultural practices and pesticides contamination level of tomato in two gardening sites in the region of Boucle. Afr. J. Agric. Res..

[42] Tankiewicz M. (2019). Determination of selected priority pesticides in high water fruits and vegetables by modified QuEChERS and GC-ECD with GC-MS/MS confirmation. Molecules.

[43] Rouamba S.S., Tapsoba F., Bazié B.S.R., Ollo Y., Kabré E., Sangaré L., Savadogo A. (2021). Assessment of the contamination of *Lactuca sativa L*. (lettuce) and *Lycopersicon esculentum* (tomato) by pesticides: Case of market gardeners in Ouagadougou. Int. J. One Health.

[44] Kpoda S., Bande M., Compaore A.M., Bazie R.B.S., Meda R.N., Somda S., Meda D.S., Kpoda H.B.N., Some S.A., Sakana L. (2020). Nutritional, microbiological, and toxicological quality assessment of foods sold in urban and suburban markets in Burkina Faso’. Health Secur..

[45] European Commission Health & Consumer Protection Directorate-General Guidance Document on Analytical Quality Control and Validation Procedures for Pesticide Residues Analysis in Food and Feed. 2013. SANCO/12571/2013. http://www.eurl-pesticides.eu/library/docs/allcrl/AqcGuidance_Sanco_2013_12571.pdf.

